# Effect of supplementation with *Lactobacillus rhamnosus* GG powder on intestinal and liver damage in broiler chickens challenged by lipopolysaccharide

**DOI:** 10.3389/fmicb.2024.1466274

**Published:** 2024-10-29

**Authors:** Xiaohan Zhang, Lanyuan Sun, Mengjun Wu, Chenmin Yu, Di Zhao, Lei Wang, Zhengfan Zhang, Dan Yi, Yongqing Hou, Tao Wu

**Affiliations:** Hubei Key Laboratory of Animal Nutrition and Feed Science, Wuhan Polytechnic University, Wuhan, China

**Keywords:** *Lactobacillus rhamnosus* GG, broiler chicken, lipopolysaccharide, intestinal health, liver

## Abstract

This study explores the effect of dietary along with *Lactobacillus rhamnosus* GG (LGG) powder on intestinal and liver damage in broiler chickens challenged by lipopolysaccharide (LPS). A total of 100 healthy 1-day-old Ross 308 broiler chickens were selected and randomly divided into two treatments: the control group and the LGG treatment group. There were five replicates for each group, with 10 chickens per replicate. The chickens in the control group were fed a basal diet, while LGG treatment was supplemented with 1,000 mg/kg LGG along with the basal diet. The experiment lasted 29 days, and the trial included two phases. During the first 27 days, the animals were weighed on the 14th and 27th days to calculate growth performance. Then, on day 29, 2 animals from each replicate were intraperitoneally injected with 1 mg/kg BW LPS, and another 2 animals were treated with an equal volume of saline. The chickens were slaughtered 3 h later for sampling and further analysis. (1) LGG addition to the diet did not affect growth performance, including average daily gain (ADG), average daily feed intake (ADFI), and feed-to-weight ratio (F/G) of broiler chickens; (2) LPS stimulation decreased villus height (VH), and caused oxidative stress and increased the amount of diamine oxidase (DAO) in plasma, and the relative expression of intestinal inflammation genes (*interleukin-8* [*IL-8*], *interleukin 1β* [*IL-1β*], *inducible nitric oxide synthase* [*iNOS*], and *tumor necrosis factor-α* [*TNF-α*]) and the relative expression of liver injury genes (*b-cell lymphoma 2* [*BCL2*], *heat shock protein70* [*HSP70*], and *matrix metallopeptidase 13* [*MMP13*]). (3) Supplementation of LGG increased VH and the relative expression of intestinal barrier genes (*mucins 2* [*Mucin2*] and *occludin* [*Occludin*]) and decreased the amount of DAO in plasma and the relative expression of intestinal inflammatory factors (*IL-8*, *iNOS*, and *IL-1β*). LGG supplementation also increased the expression of liver injury-related genes (*MMP13* and *MMP9*). In conclusion, LGG enhanced intestinal barrier function, improved intestinal morphology, and alleviated the intestines’ inflammatory response in LPS-stimulated broiler chicken, and it has a slightly protective effect on liver damage.

## 1 Introduction

Various factors such as infection, pathogenic microorganisms, and environmental pollution can cause immune stress and oxidative stress in livestock and poultry, which may threaten the health of livestock and poultry (Li et al., 2015; Yang et al., 2011). Lipopolysaccharide (LPS), a component of the Gram-negative bacteria’s cell wall, is one of the main factors causing intestinal mucosal injury (Hadfield et al., 2018). It can lead to acute inflammatory reactions and imbalances in the body’s oxidation and antioxidant defense systems, thereby promoting reactive oxygen species (ROS), causing lipid peroxidation in the body and oxidative damage to DNA and proteins (Takahashi et al., 2008; Heo et al., 2013; Zheng et al., 2016; Suliman et al., 2004). Under this condition, the body requires more energy to maintain body metabolism and the transport and absorption of proteins and amino acids, so the levels of triglyceride (TG), total cholesterol (TC), high-density liptein cholesterol (HDL) and low-density lipoprotein cholesterol (LDL) in the blood will increase (Liang et al., 2019; He et al., 2017). LPS interacts with the host and alters gene expression through intracellular signaling cascades, including nuclear factor-kappa B (NF-κB), the most critical downstream signaling pathway mediated by LPS (Xie et al., 2022; Huang et al., 2022).

The intestine is not only the central place for digestion and absorption but also the largest immune organ of the body, which can effectively prevent harmful substances such as external pathogens from invading the body The damage of the intestinal epithelial barrier increases intestinal permeability and leads to the invasion of the internal environment by adverse external factors (Pácha, 2000). The damage to the intestinal epithelial barrier increases intestinal permeability and leads to the invasion of the internal environment by adverse external factors (Gonçalves et al., 2018; Blackhurst and Funk, 2023). The gut microbial ecosystem is a collection of host-microbiota interactions and interactions between bacterial-dominated microbiota, and the gut microbiota is now considered one of the critical factors regulating host health (de Vos et al., 2022; Schiffrin and Blum, 2002). Probiotics play a crucial role in regulating the intestinal microecology of poultry (Shi et al., 2023). *Lactobacillus rhamnosus* GG (LGG) is a Gram-positive facultative anaerobic bacterium first extracted from a healthy human gastrointestinal tract in 1983 (Goldstein et al., 2015). LGG can attach to intestinal epithelial cells and form a biological barrier on the surface of the digestive tract, thereby reducing the relative abundance of *Odoribacter*, *Proteobacteria*, *Deferribacteres*, etc., and increasing the relative abundance of *Alloprevotella*, *Bacteroides*, *Parabacteroides*, *Ruminococcus*, etc.; thus promoting host defense, preventing the invasion of harmful bacteria, enhancing host immunity and improving digestive tract function (Tsai et al., 2013; Lebeer et al., 2010; Zhang et al., 2021; Wu et al., 2021; Yan et al., 2017). Studies have shown that LGG colonization of the intestine can promote the growth of microbiota, epithelial cell proliferation, differentiation, tight junction formation, IgA production and maturation during development, and reduce the risk of intestinal injury and colitis in adult mice (Donato et al., 2010). LGG has been shown to mitigate the effects of TNF-*α* on epithelial barrier integrity and inflammation (Turner, 2009). LGG has been shown to play an essential role in preventing inflammation-induced damage and promoting intestinal barrier function, which is composed of a group of protein complexes, including Occludens (ZO), Occludin, and Claudin family members (Yan and Polk, 2002; Chen et al., 2016; Han et al., 2019). Oral supplementation of *L. rhamnosus* improves the composition of the cecal microbiota of broiler chickens. It elevates the transcription of tight junction proteins in the duodenum and jejunum, thereby suppressing the gene expression of intestinal proinflammatory cytokines and promoting digestive capacity. In addition, *L. rhamnosus* can attenuate *Caspase-3* and *Caspase-9* gene expression levels, thereby attenuating intestinal cell apoptosis (Al-Khalaifa et al., 2019).

The LGG has been widely used in medical research and breeding production, but there are few reports on LGG’s resistance to immune stress. Our research group has previously established an immune stress and intestine injury model of LPS-stimulated broiler chickens. Therefore, this study aimed to explore the protective effect of LGG on the intestines and liver of broiler chickens stimulated by LPS.

## 2 Materials and methods

### 2.1 Experiment design

Remarkably, 100 healthy 1-day-old Ross 308 broiler chickens were randomly divided into two treatment groups: a control group and an LGG group, with 5 replicates in each group and 10 chickens in each replicate. The control group of broilers was fed with basic feed, while the LGG group was supplemented with LGG at a dose of 1,000 mg/kg in the basic feed. The experiment lasted for 29 days and was divided into two stages. Experiment 1: The first stage of the experiment lasted for 27 days and was weighed on the 14th and 27th days, respectively, to record the daily feed intake.

Experiment 2: the second stage lasted for 2 days, from day 27 to day 29 0.4 chickens with similar weights were selected from each replicate, 2 of which were injected with physiological saline and 2 with LPS, with an injection dose of 1 mg/kg BW. Three hours after the LPS injection, and then the chickens were killed by cervical dislocation.

### 2.2 Breeding management

All animal procedures used in the present study were approved by the Institutional Animal Care and Use Committee of Wuhan Polytechnic University (Index number: WPU202306002). Broiler chickens are raised in cages, with 10 chickens per cage. Before the start of the experiment, the chicken houses, feed troughs, chicken pens, and water troughs were thoroughly cleaned, and the chicken houses were fumigated and disinfected with potassium permanganate and formaldehyde. During the raising period, the light is maintained continuously, and the overall humidity of the chicken house is controlled at 50–60%. The temperature was controlled at 30–33°C 1 week before the chickens were introduced, then the temperature was reduced by 2–3°C every week, and the temperature was controlled at 24°C until the end of the experiment. During the experiment, the feces should be cleaned regularly and ventilated frequently, and environmental sanitation should be also maintained. The broilers’ mental status and feed intake should be observed and recorded daily.

Lipopolysaccharide (sourced from *Escherichia coli* 0111: B4, item number L2630), purchased from Sigma Reagent Company in the United States. The freeze-dried *L. rhamnosus* GG powder contained 3 × 10^9^ colony forming units (CFU)/g and was stored in the sealed packet at 4°C until used. In the experiment, the basic feed was prepared according to the Chinese broiler breeding standard (2004), and the feed composition and nutrition level are shown in Table 1.

**Table 1:** Composition and nutrient levels of basal diets (air-dry basis) (%).

Feed composition	Day 1–day 29	Nutrition level	Day 1–day 29
Corn	51.73	DM	88.50
Soybean meal	40.73	CP	20.78
Soybean oil	3.36	ADF	5.90
CaHPO_4_	1.92	NDF	11.24
Limestone	1.16	GE (MJ/kg)	24.52
NaCl	0.35	Ash	15.79
*DL*-Met	0.26	ME (MJ/kg)	2.92
Choline chloride (50%)	0.25	Ca	1.00
Mineral premix^1^	0.20	AP	0.45
Vitamin premix^2^	0.04	Lys	1.17
Total	100.00	Met + Cys	0.90
		Thr	0.82

^1The mineral premix provided the following per kg of diets: Cu, 8 mg; Zn, 75 mg; Fe, 80 mg; Mn, 100 mg; Se, 0.15 mg; and I, 0.35 mg. 2The vitamin premix provided the following per kg of diets: VD3, 2,500 IU; VE12, 0.025 mg; VB1, 2 mg; VB2, 2 mg; VE, 30 IU; D-biotin, 0.0325 mg; folic acid, 1.25 mg; pantothenic acid, 12 mg. DM, dry matter; CP, crude protein; ADF, acid detergent fiber, NDF, neutral detergent fiber; GE, gross energy; and Ash are measured values, while the others were calculated values; AP, Available Phosphorus; Ca, Calcium.^

### 2.3 Samples collection

Approximately 3 h after the LPS injection, blood was collected from the broiler chickens. Fabricius’s duodenum, jejunum, ileum, liver, thymus, spleen, and bursa were quickly dissected and separated, and their weights were recorded. Each intestine was rinsed with precooled PBS, frozen in liquid nitrogen, and then stored at −80°C for a long period until testing. A sample of about 1 cm from each intestine was fixed in a 4% paraformaldehyde solution and sent to Wuhan Bolfu Biochemical Company for hematoxylin and eosin (H&E) staining sections.

### 2.4 Growth performance

Based on recorded body weight and feed intake, the average daily gain (ADG), average daily feed intake (ADFI), and feed-to-weight ratio (F/G) were calculated.

### 2.5 Blood biochemical measurements

The collected blood was separated into plasma and stored at −80°C. Concentrations of biochemical parameters in plasma [total bilirubin (TB), total cholesterol (TC), triglyceride (TG), albumin (ALB), alanine aminotransferase (ALT), alkaline phosphatase (ALP), *γ*-glutamyl transpeptidase (GGT), lactate dehydrogenase (LDH), high-density lipoprotein (HDL) cholesterol, low-density lipoprotein (LDL) cholesterol] were measured with corresponding kits using a Hitachi 7,060 Automatic Biochemical Analyzer. (Hitachi, Japan).

### 2.6 Organ index

Fabricius’s liver, thymus, spleen, and bursa were weighed and recorded. Organ index (g/kg) = fresh weight of the organ (g)/fasting live weight of the broiler before slaughter (kg).

### 2.7 Plasma DAO index

The diamine oxidase (DAO) amount detection kit was purchased from Nanjing Jiancheng Bioengineering Institute. All procedures were followed by the kit instructions (DAO: Category number A088-1-1).

### 2.8 Plasma antioxidant enzymes and concentrations of oxidation-relevant products

Fractionated plasma was used to analyze antioxidant and oxidation-related products. The activities of catalase (CAT), total superoxide dismutase (T-SOD), myeloperoxidase (MPO), and the concentration of hydrogen peroxide (H_2_O_2_) and malondialdehyde (MDA) were determined by using commercially available kits (Nanjing Jiancheng Bioengineering Institute, Nanjing, China), according to the protocols of the manufacturer.

### 2.9 Intestinal tissue morphology

The intestinal segments were removed from the 4% paraformaldehyde solution and embedded in paraffin, and H&E staining sections were made. Randomly select 5–10 relatively complete intestinal villi from the slice images, and use an OLYMPSBX-41TF optical microscope (BX-41, Olympus, Japan) to observe and measure the villus height (VH) and crypt depth (CD). VH is the vertical distance from the top of the villus to the opening of the intestinal gland; CD is the vertical distance from the crypt opening to the crypt bottom.

### 2.10 Quantitative RT-PCR assay

Total RNA from intestinal or liver tissue was extracted using TRIzol™ Reagent Kit (Invitrogen, United States). Approximately 0.1 g intestinal or liver tissue in a 2-ml Eppendorf Tube (EP) was added with a 1-ml TRIzol Reagent, then homogenized at 4°C. Total RNA was extracted according to the instructions by the kit’s manufacturer. The RNA concentration and purity of the sample (A260:A280 = 1.7–2.1, A260:A230 > 2.0) were determined using a microspectrophotometer (NanoDrop 2000, Thermo Fisher Scientific, Inc., United States), and all Samples were diluted evenly to the same concentration. The extracted RNA was reverse transcribed into cDNA using PrimeScript^®^ RT Reagent Kit with gDNA Eraser (TaKaPa, Dalian). SYBR^®^ Premix Ex TaqTM (TaKaPa, Dalian) on-machine kit was used to detect the expression of genes related to intestinal barrier function, tissue injury, and inflammatory response through a real-time fluorescence quantitative polymerase chain reaction (PCR) instrument (ABI 7500, Thermo Fisher Scientific, United States). *β*-actin was used as the internal reference gene. The sequences of the primers used in the experiment are shown in Table 2. The primers were synthesized by Shanghai Sangon Bioengineering Co., Ltd. PCR reaction system 10 μL: Tap Pro Universal SYBR qPCR Master Mix 5 μL, cDNA 1 μL (50 ng), RNase-free ddH_2_O 3.6 μL, upstream and downstream primers (10 μmol/L) 0.2 μL each. PCR amplification program: predenaturation at 95°C for 30 s; denaturation at 95°C for 5 s, annealing at 60°C for 34 s, a total of 40 cycles; melting curve program: denaturation at 95°C for 15 s, annealing at 60°C for 1 min, and extension at 95°C for 15 s. The relative expression of each gene was calculated using the 2^−ΔΔCt^ method.

**Table 2:** Primer sequences.

Gene name	Sequences (5′–3′)	Product	Accession numbers
*HSP70*	F: TCTCATCAAGCGTAACACCAC R: TCTCACCTTCATACACCTGGAC	104	AY143693.1
*IFN-γ*	F: AGCTGACGGTGGACCTATTATT R: GGCTTTGCGCTGGATTC	259	NM_205149.2
*IL-1β*	F: ACTGGGCATCAAGGGCTA R: GGTAGAAGATGAAGCGGGTC	256	NM_204524.2
*IL-8*	F: ATGAACGGCAAGCTTGGAGCTG R: TCCAAGCACACCTCTCTTCCATCC	233	AJ009800.1
*iNOS*	F: CAGCTGATTGGGTGTGGAT R: TTTCTTTGGCCTACGGGTC	158	U46504.1
*MMP13*	F: GTGCTTCCTGATGATGATGTGC R: TCGCCAGAAAAACCTGTCCTT	258	NM_001293090.2
*MMP9*	F: ATGAACTACTCCCCCGACCTG R: AGTCCAGAACTCATCATCATCG	258	NM_204667.2
*Mucin-2*	F: TTCATGATGCCTGCTCTTGTG R: CCTGAGCCTTGGTACATTCTTGT	93	XM_040701656.2
*Occludin*	F: ACGGCAGCACCTACCTCAA R: GGGCGAAGAAGCAGATGAG	123	NM_205128.1
*TNF-α*	F: GAGCGTTGACTTGGCTGTC R: AAGCAACAACCAGCTATGCAC	64	NM_204267.2
*BCL2*	F: CACCTGGATGACCGAGTACC R: GTCCAAGATAAGCGCCAAGA	191	NM_205339.2
*CAT*	F: GGTTCGGTGGGGTTGTCTTT R: CACCAGTGGTCAAGGCATCT	211	NM_001031215.2
*GSH1*	F: GACCAACCCGCAGTACATCA R: GAGGTGCGGGCTTTCCTTTA	205	NM_001277853.2
*SOD*	F: GGTGCTCACTTTAATCCTG R: CTACTTCTGCCACTCCTCC	109	NM_205064.1
*β-actin*	F: GAGAAATTGTGCGTGATCA R: CCTGAACCTCTCATTGCCA	152	NM_205518.2

β-actin, β-actin; BCL2, b-cell lymphoma 2; CAT, catalase; GSH1, glutathione 1; HSP70, heatshockprotein70; IFN-γ, interferon gamma; IL-1β, interleukin 1 beta; IL-8, interleukin-8; iNOS, inducible nitric oxide synthase; MMP13, matrix metallopeptidase 13; MMP9, matrix metalloproteinase-9; Mucin-2, mucins 2; Occludin, occludin; SOD, superoxide dismutase; TNF-α, tumor necrosis factor-α.

### 2.11 Statistical analysis

The experimental data were analyzed using Statistical Package for the Social Sciences (SPSS) version 27.0 (SPSS, Inc., Chicago, IL, United States) statistical software. Growth performance data were assessed using one-way analysis of variance (ANOVA) followed by Duncan’s multiple comparisons test. The remaining data were analyzed using a 2 × 2 factorial design followed by Duncan’s multiple comparisons test. Among them, *p* < 0.05 denotes the difference is significant, and 0.05 ≤ *p* ≤ 0.10 denotes a trend.

## 3 Results

### 3.1 The effect of LGG on the growth performance of broiler chickens

As shown in Table 3, LGG addition to the diet did not have a significant impact on ADG, ADFI, and F/G of broiler chickens.

**Table 3:** Effect of LGG on the growth performance of broiler chickens.

Items	Control	LGG group	*p-*value
Initial weight (g)	41.63		
D14 weight (g)	425.18 ± 5.73	431.18 ± 10.29	0.288
D27 weight (g)	1217.73 ± 27.96	1230.66 ± 54.05	0.647
1–14 days of age			
ADG (g/d)	27.40 ± 0.41	27.83 ± 0.74	0.286
ADFI (g/d)	34.42 ± 0.5	34.67 ± 1.51	0.742
F/G	1.26 ± 0.02	1.25 ± 0.03	0.578
14–27 days of age			
ADG (g/d)	60.96 ± 2.35	61.50 ± 3.85	0.797
ADFI (g/d)	92.60 ± 2.95	92.05 ± 4.39	0.822
F/G	1.52 ± 0.03	1.50 ± 0.03	0.339
1 to 27 days of age			
ADG (g/d)	44.18 ± 1.08	44.66 ± 2.06	0.656
ADFI (g/d)	63.52 ± 1.62	63.36 ± 2.62	0.913
F/G	1.44 ± 0.02	1.42 ± 0.02	0.197

Data are mean ± standard deviation (SD), *n* = 10. ADG, average daily gain; ADFI, average daily feed intake; F/G, feed-to-weight ratio; LGG, *Lactobacillus rhamnosus* GG.

### 3.2 The effect of LGG on plasma biochemical indicators in LPS-stimulated broiler chickens

As listed in Table 4, LPS stimulation increased the ALT activity and decreased the amounts of TC, TG, ALB, ALP, HDL, and LDL in plasma, while LGG increased the amount of LDL and ALT activity (*p* < 0.05). There was a tendency for LGG to increase the amount of TB (*p* = 0.071).

**Table 4:** Effect of LGG on plasma biochemical indicators in LPS-stimulated broiler chickens.

Items	−LPS		+LPS		SEM	*p*-value		
	−LGG	+LGG	−LGG	+LGG		LPS	LGG	LPS × LGG^a,b^
TB (mg/dl)	0.35	0.41	0.36	0.38	0.068	0.528	0.071	0.364
TC (mg/dl)	119.58	119.39	111.56	100.06	14.667	0.002	0.156	0.170
TG (mg/dl)	21.220	20.76	16.30	14.92	5.676	0.002	0.576	0.779
ALB (g/dl)	16.28	15.11	14.58	13.76	2.369	0.040	0.172	0.805
ALT (U/L)	2.60	3.20	3.00	4.40	1.265	0.029	0.007	0.262
ALP (U/L)	3219.14	3242.86	2749.44	2531.43	730.128	0.030	0.709	0.643
GGT (U/L)	15.50	16.40	16.11	17.70	3.218	0.364	0.239	0.742
LDH (mg/dl)	0.07	0.09	0.08	0.08	0.021	0.646	0.184	0.313
HDL (mg/dl)	108.05	112.16	102.6	99.59	11.526	0.014	0.876	0.313
LDL (mg/dl)	15.34	23.12	13.01	19.15	5.483	0.021	<0.001	0.532

^a,b^Different alphabets denote differences in significant difference (*p* < 0.05). Data are mean ± scanning electron microscope (SEM), *n* = 10. ALB, albumin; ALP, alkaline phosphatase; ALT, alanine aminotransferase; GGT, γ-glutamyl transpeptidase; HDL, high-density lipoprotein cholesterol; LDH, lactate dehydrogenase; LDL, low-density lipoprotein cholesterol; LGG, *Lactobacillus rhamnosus* GG; LPS, lipopolysaccharide; TB, total bilirubin; TC, total cholesterol; TG, triglyceride.

### 3.3 The effect of LGG on antioxidant enzymes and concentrations of oxidation-relevant products in LPS-stimulated broiler chickens

As shown in Table 5, LPS and LGG significantly decreased the GSH-Px activity in plasma (*p* < 0.001). On the contrary, there were tendencies that LPS decreased T-SOD activity (*p* = 0.065) and H_2_O_2_ content (*p* = 0.098).

**Table 5:** Effect of LGG on plasma antioxidant indicators in LPS-stimulated broiler chickens.

Items	−LPS		+LPS		SEM	*p-*value		
	−LGG	+LGG	−LGG	+LGG		LPS	LGG	LPS × LGG^a–c^
MDA (nmol/ml)	1.36	1.50	1.62	1.49	0.095	0.189	0.928	0.159
T-SOD (U/ml)	407.44	393.50	367.22	357.84	19.942	0.065	0.562	0.910
H_2_O_2_ (nmol/L)	21.65	22.21	21.56	16.50	1.709	0.098	0.196	0.109
GSH-Px (U/mg of protein)	3063.51	2421.76	2313.49	1487.23	140.276	<0.001	<0.001	0.515

^a–c^Different alphabets denote differences in significant difference (*p* < 0.05). Data are mean ± scanning electron microscope (SEM), *n* = 10. GSH-Px, glutathione peroxidase; H_2_O_2_, hydrogen peroxide; LGG, *Lactobacillus rhamnosus* GG; LPS, lipopolysaccharide; MDA, malondialdehyde; T-SOD, total superoxide dismutase.

### 3.4 The effect of LGG on plasma DAO index of LPS-stimulated broiler chickens

**Figure 1:**
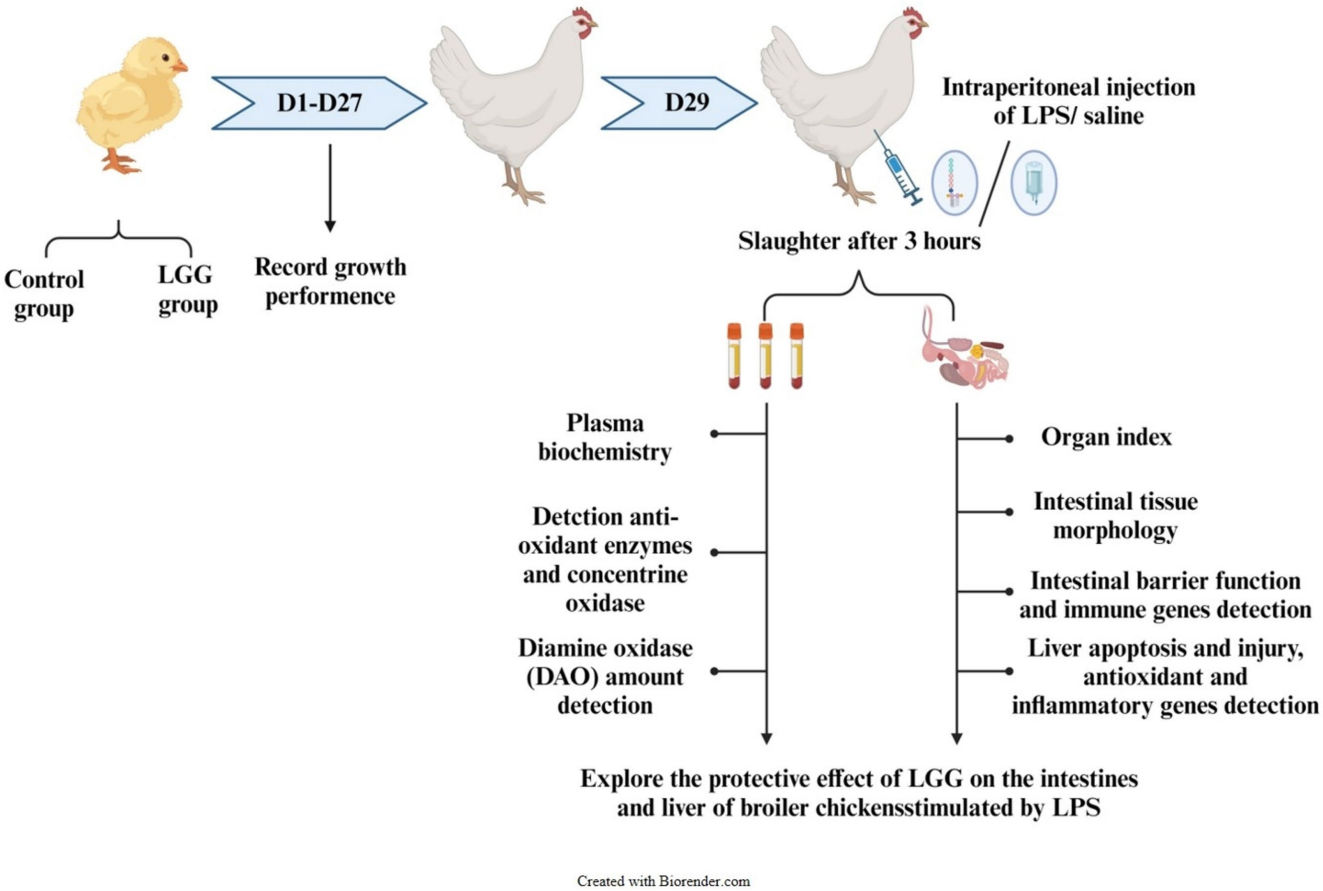
Experimental design route. DAO, diamine oxidase; LGG, *Lactobacillus rhamnosus* GG; LPS, lipopolysaccharide.

As shown in Figure 1, LPS (*p* = 0.036) and LGG (*p* < 0.001) significantly decreased the amount of DAO in plasma. There were interactive effects on the amount of plasma. Under LPS stimulation, the supplementation of LGG decreased the amount of DAO in plasma (*p* < 0.001).

### 3.5 The effect of LGG on intestinal tissue morphology of LPS-stimulated broiler chickens

**Figure 2:**
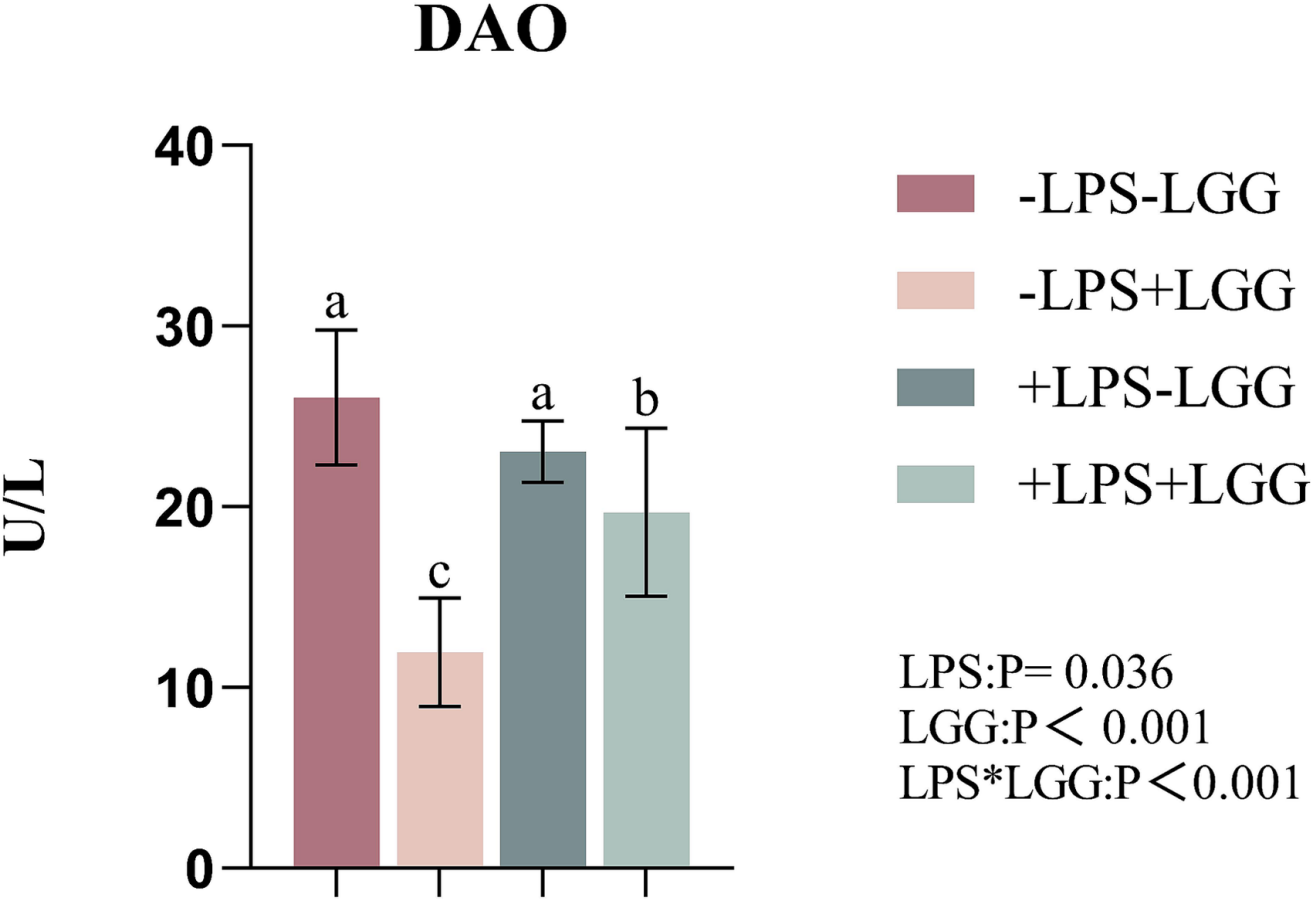
Effect of LGG on the plasma DAO index of LPS-stimulated broiler chickens. Data are mean ± standard deviation (SD), *n* = 10. a, b, and c: Different alphabets denote differences in significant difference (*p* < 0.05). DAO, diamine oxidase; LGG, *Lactobacillus rhamnosus* GG; LPS, lipopolysaccharide; MDA.

**Figure 3:**
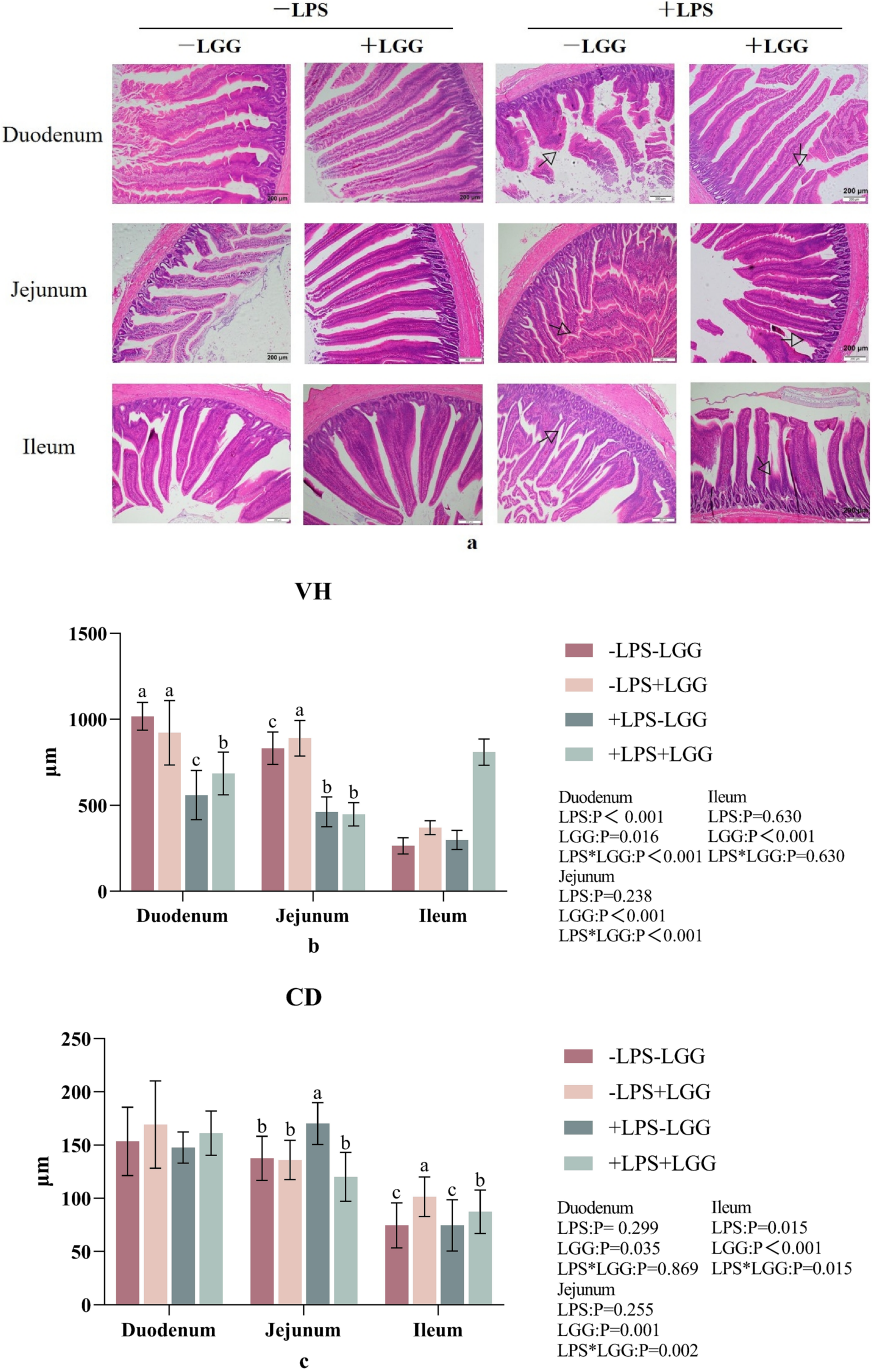
Effect of LGG on intestinal tissue morphology in LPS-stimulated broiler chickens. Data are mean ± standard deviation (SD), *n* = 10. **(a)** Intestinal morphology HE section. “
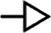
” marked the site of villous damage. **(b)** Villus height (VH). **(c)** Crypt depth (CD). a, b, and c: Different alphabets denote differences in significant difference (*p* < 0.05). LGG, *Lactobacillus rhamnosus* GG; LPS, lipopolysaccharide.

As shown in Figure 2, alphabet a: LPS stimulation caused the intestinal villi to fall off or break in broiler chickens, and the integrity of the villi is reduced, while the LGG addition maintained the structural integrity of the villi to a certain extent; alphabets b and c: LPS stimulation significantly decreased VH in duodenum (*p* < 0.001), CD in ileum (*p* = 0.015). LGG addition increased VH in the duodenum (*p* = 0.016), jejunum (*p* < 0.001) and ileum (*p* < 0.001), CD in the duodenum (*p* = 0.035), and ileum (*p* < 0.001). There were interactive effects on CD in the jejunum and ileum. Compared with broiler chickens stimulated with LPS, LGG addition significantly increased VH (*p* < 0.001) in the duodenum and CD in the ileum (*p* = 0.015) and decreased CD in the jejunum (*p* = 0.002) (Figure 3).

### 3.6 The effect of LGG on the expression of genes related to LPS-stimulated intestinal barrier function in broiler chickens

As shown in Table 6, LPS significantly elevated the relative expression of *Occludin* in the jejunum (*p* = 0.014) but decreased the relative expression of *Mucin2* in the duodenum (*p* = 0.001). LGG significantly increased the relative expression of *Mucin2* in the duodenum (*p* < 0.001) and *Occludin* in the jejunum (*p* < 0.001). There were interactive effects on the relative expression of *Occludin* in the duodenum and *Mucin2* in the jejunum of broiler chickens. Compared with broiler chickens stimulated with LPS, the relative expression of *Mucin2* in the jejunum (*p* < 0.001) of broiler chickens was higher after LGG supplemented.

**Table 6:** Effect of LGG on the expression of genes related to LPS-stimulated intestinal barrier function in broiler chickens.

Items	−LPS		+LPS		SEM	*p*-value		
	−LGG	+LGG	−LGG	+LGG		LPS	LGG	LPS × LGG
Duodenum								
*Mucin2*	1.00	1.40	0.68	1.05	0.061	0.001	<0.001	0.889
*Occludin*	1.00^a^	0.85^ab^	0.75^b^	0.978^ab^	0.040	0.443	0.629	0.020
Jejunum								
*Mucin-2*	1.00^a^	0.76^b^	0.67^b^	0.965^a^	0.038	0.334	0.664	<0.001
*Occludin*	1.00	2.03	1.25	2.53	0.120	0.014	<0.001	0.385

^a,b^Different alphabets denote differences in significant difference (*p* < 0.05). Data are mean ± scanning electron microscope (SEM), *n* = 10. LGG, *Lactobacillus rhamnosus* GG; LPS, lipopolysaccharide.

### 3.7 The effect of LGG on LPS-stimulated organ index in broiler chickens

As shown in Table 7, There is an interaction between LPS stimulation and LGG. Under LPS stimulation, LGG addition can significantly increase the relative weight of the bursa of Fabricius in broilers (*p* = 0.006).

**Table 7:** Effect of LGG on the relative weight (%) of LPS-stimulated organs in broiler chickens.

Items	−LPS		+LPS		SEM	*p*-value		
	−LGG	+LGG	−LGG	+LGG		LPS	LGG	LPS × LGG
Liver	24.21	24.08	24.79	26.41	4.962	0.644	0.372	0.590
Spleen	0.95	0.88	0.86	0.92	0.247	0.786	0.921	0.432
Thymus	3.63	3.72	2.89	3.38	1.136	0.136	0.429	0.579
Bursa of Fabricius	2.29^a^	1.79^ab^	1.36^b^	2.12^a^	0.747	0.168	0.542	0.006

^a,b^Different alphabets denote differences in significant difference (*p* < 0.05). Data are mean ± scanning electron microscope (SEM), *n* = 10. LGG, *Lactobacillus rhamnosus* GG; LPS, lipopolysaccharide.

### 3.8 The effect of LGG on intestinal inflammation in broiler chickens stimulated with LPS

As listed in Table 8, LPS stimulation significantly elevated the relative expression of *IL-8*, *IL-1β*, and *TNF-α* in the duodenum (*p* < 0.001), and elevated the relative expression of *IL-8*, *IL-1β*, *iNOS*, and *TNF-α* in the jejunum (*p* < 0.001). LGG addition significantly decreased the relative expression of *IL-8* (*p* = 0.038) *iNOS* (*p* < 0.001) in the duodenum, and *IL-1β* (*p* = 0.027) in the Jejunum; LGG addition significantly increased the relative expression of *IL-1β* (*p* = 0.014) and *TNF-α* (*p* = 0.010) in the duodenum and *IL-8* (*p* = 0.001), and *TNF-α* (*p* < 0.001) in the Jejunum. There is an interaction between LPS stimulation and LGG. Under LPS stimulation, LGG addition can significantly increase the relative expression of *TNF-α* in the jejunum (*p* = 0.001).

**Table 8:** Effect of LGG on the expression of inflammation-related genes in the gut of LPS-stimulated broiler chickens.

Items	−LPS		+LPS		SEM	*p*-value		
	−LGG	+LGG	−LGG	+LGG		LPS	LGG	LPS × LGG
Duodenum								
*IL-8*	1.00	0.61	1.95	1.78	0.111	<0.001	0.038	0.400
*IL-1β*	1.00	1.16	1.94	2.62	0.135	<0.001	0.014	0.111
*iNOS*	1.00	0.25	1.10	0.32	0.073	0.212	<0.001	0.800
TNF-α	1.00	1.30	1.78	2.37	0.118	<0.001	0.010	0.371
Jejunum								
*IL-1β*	1.00	0.89	2.59	1.99	0.135	<0.001	0.027	0.122
*IL-8*	1.00	2.10	2.86	3.36	0.178	<0.001	0.001	0.190
*iNOS*	1.00	0.84	1.43	1.38	0.060	<0.001	0.265	0.545
*TNF-α*	1.00^b^	1.11^b^	1.31^b^	2.20^a^	0.093	<0.001	<0.001	0.001

^a,b^Different alphabets denote differences in significant difference (*p* < 0.05). Data are mean ± scanning electron microscope (SEM), *n* = 10. LGG, *Lactobacillus rhamnosus* GG; LPS, lipopolysaccharide.

### 3.9 The effect of LGG on liver function in broiler chickens stimulated with LPS

As shown in Table 9, LPS significantly increased the relative expression of IL-8, *IL-1β*, *iNOS*, and *TNF-α* (*p* < 0.001) in the liver of broiler chickens.

**Table 9:** Effect of LGG on LPS-stimulated liver function-related genes in broiler chickens.

Items	−LPS		+LPS		SEM	*p*-value		
	−LGG	+LGG	−LGG	+LGG		LPS	LGG	LPS × LGG
Apoptosis and injury gene								
*HSP70*	1.00	1.04	0.58	0.66	0.046	<0.001	0.397	0.782
*BCL2*	1.00	1.01	0.72	0.89	0.036	0.004	0.185	0.258
*MMP9*	1.00^c^	1.00^c^	3.12^a^	2.27^b^	0.164	<0.001	0.013	0.013
*MMP13*	1.00	1.25	0.36	0.60	0.066	<0.001	0.003	0.949
Antioxidant genes								
*CAT*	1.00	1.02	0.54	0.33	0.055	<0.001	0.105	0.060
*GSH1*	1.00	1.00	1.29	1.55	0.062	<0.001	0.234	0.221
*SOD*	1.00	0.99	0.74	0.90	0.037	0.013	0.289	0.204
Inflammatory genes								
*IL-1β*	1.00	1.31	3.71	3.78	0.236	<0.001	0.421	0.599
*IL-8*	1.00	0.73	7.52	8.82	0.627	<0.001	0.253	0.085
*iNOS*	1.00	0.95	27.44	32.36	2.457	<0.001	0.138	0.129
*TNF-α*	1.00	0.98	2.85	2.81	0.168	<0.001	0.847	0.954

^a,b^Different alphabets denote differences in significant difference (*p* < 0.05). Data are mean ± scanning electron microscope (SEM), *n* = 10.

As listed in Table 9, LPS stimulation significantly decreased the relative expression of *BCL2* (*p* = 0.004), *HSP70* (*p* < 0.001), and *MMP13* (*p* < 0.001) in the liver and increased the relative expression of *MMP9* (*p* < 0.001); LGG addition significantly increased the relative expression of *MMP13* (*p* = 0.003) and *MMP9* (*p* = 0.013). There is an interaction between LGG and LPS stimulation in the relative expression of *MMP9*. Compared with broiler chickens stimulated with LPS, LGG addition significantly elevated the relative expression of *MMP9* in the liver (*p* = 0.013).

As shown in Table 9, LPS stimulation significantly decreased the relative expression of *SOD* (*p* = 0.013) and *CAT* (*p* < 0.001) in the liver but significantly elevated the relative expression of *GSH1* (*p* < 0.001) in the liver.

## 4 Discussion

### 4.1 Effect of LGG on the growth performance of broiler chickens

The beneficial effects of LGG in animal production have been confirmed by numerous experiments. Probiotics can effectively promote the absorption of mineral elements, vitamin metabolism, and physiological regulation of the digestive tract, thereby improving the growth performance of animals (Zhang et al., 2024). Studies have shown that LGG addition can significantly decrease the number of *E. coli* in the intestinal tract of chickens, increase ADG, reduce F/G, and improve growth performance (Chen et al., 2018). However, in this experiment, LGG had no significant effect on the growth performance of broiler chickens, and consistent results were found in the study of Song. Song et al. (2022) found that *Lactobacillus plantarum* had no significant effect on the growth performance of Daheng broilers. Song et al. (2006) found Miichthysmiiuy different doses of *Clostridium butyricum* (10^3^ ~ 10^9^ CFU/g basal diet), and the results showed that the growth performance of the fish was related to the content of *C. butyricum*. Therefore, we speculate that the lack of significance in the growth performance of this test may be related to the content of LGG.

### 4.2 The effect of LGG on blood indicators in LPS-stimulated broiler chickens

Blood has the function of transporting nutrients in the animal body, and blood indicators reflect the absorption of nutrients and the health status of the body to a certain extent. TC refers to the sum of cholesterol contained in all lipoproteins in the blood. TG and TC jointly reflect the liver’s ability to metabolize fat and nutritional status. ALB content reflects the transport of substances in the blood, cell osmotic pressure, and the body’s immune status (Zhang et al., 2021). ALT can effectively reflect liver injury; ALP can reflect bile duct damage, and both are important indicators of liver health (Aravind et al., 2003). The high amount of HDL means that the animal body can inhibit the development of diseases such as cancer by affecting immune responses, while lower HDL means that the ability of LDL to oxidize is enhanced, leading to increased intracellular oxidative stress (Ikonen, 2008). Studies have shown that LPS can significantly increase the amount of ALT in serum and significantly decrease the amount of TC, TG, ALB, ALT, HDL, and LDL in serum (Zhang et al., 2021; Huang et al., n.d.). Consistent with the results of this experiment, it shows that LPS may cause liver injury, resulting in a decrease in liver metabolism and nutritional status. The above results indicate that LPS causes liver injury, and LGG has a slight effect on the plasma parameters of broiler chickens.

### 4.3 The effect of LGG on antioxidant enzymes and concentrations of oxidation-relevant products in LPS-stimulated broiler chickens

In livestock and poultry production, oxidative stress is associated with various syndromes. Factors such as environmental, physiological, and pathological influences can easily lead to oxidative stress in the body, resulting in injury to tissue function (Li et al., 2019). The liver is an important central organ for metabolism and detoxification of the body. We have found that LPS caused liver injury and the study shows the liver is also a targeted effector organ in response to oxidative stress (Li et al., 2015). Antioxidant enzymes like T-SOD, GSH-Px, and CAT play a crucial role in scavenging free radicals in the body. The activity of these enzymes directly indicates the body’s antioxidant capacity and helps maintain the balance of free radicals (Kumar et al., 2012). GSH-Px, in particular, is a key peroxide-decomposing enzyme that can effectively eliminate superoxide and hydroxyl free radicals, safeguarding cell membrane structure and function from peroxide-induced damage (Limón-Pacheco and Gonsebatt, 2009). GSH1 encodes glutathione synthase, which is a key enzyme in the glutathione synthesis process. Glutathione has antioxidant effects (Lisowsky, 1993; Grant et al., 1997). SOD is a type of enzyme that can catalyze the dismutation of superoxide anion free radicals (O^2−^) into hydrogen peroxide (H_2_O_2_). Its main function is to remove superoxide anion free radicals that are harmful to the body. Low activity of enzymes such as SOD and GSH-Px in the blood will break the homeostasis between the production and removal of free radicals in the body (Saltiel and Olefsky, 2017). Studies have shown that LPS can significantly decrease the activities of GSH-Px, CAT, and T-SOD in serum and tissues, causing oxidative injury and liver injury (Huang et al., n.d.; Li et al., 2015; Zheng et al., 2016; Zheng et al., 2020; Han et al., 2020). This result is similar to the results of this experiment. In this experiment, LPS significantly decreased GSH-Px activity in plasma and the relative expression of CAT and SOD in the liver and had a tendency to reduce T-SOD activity in plasma. The above results indicate that LPS causes oxidative stress in the body. MA studies have found that LGG addition can increase the GSH-Px activity in the kidney and alleviate renal lipid peroxidation caused by DON (Ma et al., 2022). However, the results of this experiment show that LGG addition will significantly reduce the activity of GSH-Px in serum, which may be related to the complex redox reactions of the body. More relevant research is needed to explain it.

### 4.4 The effect of LGG on LPS-stimulated intestinal barrier function in broiler chickens

The small intestine is the main place where animals digest and absorb nutrients. The mechanical barrier is an important component of the intestinal mucosal barrier, and the tight junctions between cells and the underlying adherens junctions and desmosomes play an important role in maintaining the integrity of the intestinal mechanical barrier (Yao et al., 2022). DAO is a biomarker related to intestinal permeability, and its serum level can reflect the integrity of the intestinal barrier. DAO mainly exists in intestinal epithelial cells, and a complete intestinal mucosal barrier can prevent D-lactic acid and DAO from entering the portal circulation. When the intestinal barrier is damaged, it can enter the blood circulation system through the intestinal mucosa, resulting in increased DAO activity in the plasma (Ji et al., 2018; Fukudome et al., 2014; Xue et al., 2018). LPS not only changes the normal intestinal morphology but also damages the intestinal mechanical barrier. Studies have shown that LPS treatment will reduce the expression of tight junction proteins Occludin and ZO-1 in the ileum of piglets and increase the activity of DAO in the serum of piglets (Sun et al., 2021). Deng’s research found that *Lactobacillus casei* can significantly reduce the secretion of DAO in the intestinal tract of chickens, improve intestinal permeability, and protect the intestinal mucosal barrier function (Deng et al., 2021). In this experiment, LGG addition significantly decreased the amount of DAO, and the same results were obtained under LPS stimulation, which is consistent with Deng’s results and further illustrates that LGG may have the function of protecting the intestinal barrier.

Digestion and absorption are the most basic functions of the intestine. Different types of animals and different parts of the digestive tract have different digestion methods. The digestion and absorption functions of poultry are mostly concentrated in the small intestine (Wu et al., 2013). Whether the intestinal morphology is normal or not can reflect the digestion and absorption function of the animal’s intestines. Studies have shown that LPS can cause intestinal morphological damage, such as mucosal edema, bleeding, and even necrosis (Ma et al., 2015). Previous studies have shown that LPS stimulation destroyed the intestinal structure of broiler chickens, which is manifested by the decrease of VH and the increase of CD (Li et al., 2015; Hu et al., 2011). Consistent with the above results, in this experiment, LPS significantly decreased the VH of the duodenum. Studies have found that oral administration of various probiotic lactic acid bacteria, such as *L. rhamnosus* and *Lactobacillus reuteri* can significantly increase the VH (Ciorba et al., 2012). Mao et al. found that *L. rhamnosus* significantly increased the VH in the jejunal mucosa of weaned piglets (Mao et al., 2016). Consistent with this experiment, in this experiment, LGG addition significantly increased the VH in the duodenum, jejunum and ileum. Under LPS stimulation, LGG addition significantly increased the VH in the duodenum. In summary, it was found that LGG has the ability to alleviate the injury to the intestinal morphological structure caused by LPS, improve the digestive and absorption function of the small intestine, and protect the integrity of intestinal mucosal tissue morphology.

The tight junctions of intestinal epithelial cells are the prerequisite for ensuring the structural integrity of the intestinal mucosal barrier. Occludin is a transmembrane protein whose main function is to maintain the tight junction structure and protect the intestinal barrier (Aravind et al., 2003; Bewley et al., 2013). The mucus secreted by intestinal mucosal epithelial cells is also one of the important tissue parts of the intestinal mucosal barrier (Johansson et al., 2013). The main component of mucus is mucin, of which Mucin2 is one of the most abundant mucins secreted by goblet cells (Kurashima and Kiyono, 2017). It can form a protective mucus layer and dynamically interact with intestinal epithelial cells, microbiota, and host immune defense to maintain intestinal Mucosal homeostasis (Martens et al., 2018; Castagliuolo et al., 1996). Therefore, changes in Mucin2 are related to a variety of intestinal diseases and are an important factor in expressing the status of the intestinal mucosa. LPS stimulation reduced the expression of Mucin-2 and Occludin in the intestinal tract of broiler chickens (Cui et al., 2024; Yang et al., 2019). In this experiment, LPS significantly decreased the expression of Mucin-2 in the duodenum, consistent with the above results, but LPS significantly increased the expression of Occludin in the jejunum. It is speculated that it may be because LPS activates the autoimmune regulatory function of broilers and increases the expression of tight junction proteins to resist the injury of LPS to the intestines. Combined with the intestinal morphology data, it was considered that LPS stimulation caused damage to the intestinal barrier. Studies by Patricia WLin and others have shown that LGG can regulate the expression of genes related to intestinal epithelial cell proliferation, differentiation, and migration, induce the expression of antiapoptotic genes, maintain the stability of epithelial cells, and activate protein kinase C and mitogen-activated protein kinase pathways to promote Occludin expression in intestinal epithelial cells (Lin et al., 2008; Patel et al., 2012; Shaffiey et al., 2016). Studies have pointed out that probiotics, in addition to chickens, can significantly increase the gene expression of Occludin and Mucin2 in the ileum (Chang et al., 2020). The results of the present experiment were consistent with the above studies. In this experiment, LGG addition significantly increased the relative expression of Mucin-2 in the duodenum and significantly increased the relative expression of Occludin in the jejunum. Under LPS stimulation, LGG addition significantly increased the relative expression of Mucin-2 in the jejunum. Combining the present results that LGG decreased DAO amount in plasma and improved injury intestinal morphology, it has shown that LGG alleviated LPS-induced intestinal damage.

### 4.5 The effect of LGG on LPS-stimulated immune function in broiler chickens

The strength of the animal’s immune function and the body’s nutritional health status can be reflected by organ indexes. The spleen, thymus, and bursa of Fabricius are the main organs for immune regulation in the body (Nakamura et al., 2012; Rivas and Fabricant, 1988). The bursa of Fabricius is a central immune organ unique to birds, participating in humoral immune responses and improving resistance. Chen et al.’s study showed that 1.5 g/kg of xylo-oligosaccharide (100 mg/g), *C. butyricum* (2 × 10^9^ CFU/g), *Bacillus subtilis* (3×10^10^ CFU/g) and attapulgite Synbiotics addition can significantly increase the bursa index and intestinal immunoglobulin content of Cherry Valley ducks, suggesting that synbiotics can significantly promote the development of immune organs in Cherry Valley ducks and improve the body’s immune function (Chen et al., 2018). In this experiment, under the stimulation of LPS, LGG addition can significantly increase the relative weight of the bursa of Fabricius, which is consistent with the above results, suggesting that LGG can promote the development of immune organs in broiler chickens.

In the intestine, the homeostasis of the intestinal immune system affects intestinal and body health. Cytokines are a type of soluble small peptides secreted by immune cells and tissue cells that regulate each other between cells. IL-1β and IL-8 all belong to cells Factors can regulate the body’s immune response through receptors and play an important role in mediating trauma, infection, autoimmune diseases, and inflammatory reactions (Zhang et al., 2020). IL-8 is an important member of the chemokine family. It is secreted by macrophages and other cells. It can mediate neutrophils to participate in the body’s inflammatory response and is an important proinflammatory factor. It induces the body to produce inflammatory mediators and promote a large number of inflammatory factors (TNF-*α* and IL-8) (Hsieh et al., 2018; Ren et al., 2021). iNOS is mainly expressed in giant cells and leukocytes. In inflammatory reactions, iNOS promotes the release of NO (Hashemi Goradel et al., 2019; Anavi and Tirosh, 2020; Lopes et al., 2022). LPS significantly decreases the expression of *Occludin* and *Claudin-1* and mediates the expression of *IL-1β*, *IL-8*, and *TNF-α* inflammatory cytokines through the NF-κB and Nrf2 signaling pathways. The expression of inflammatory mediators can mediate iNOS, and excessive production of NO induces oxidative stress, accelerates inflammatory reactions, and leads to intestinal injury (Zhang et al., 2020; Song et al., 2016; Xu et al., 2023; Han et al., 2017). In this experiment, LPS significantly increased the relative expression of *IL-8*, *IL-1β*, and *TNF-α* in the duodenum and *IL-1β*, *IL-8*, *iNOS*, and *TNF-α* in the jejunum and liver. The results are the same as the above, indicating that we caused an inflammatory response in the intestine through LPS, and the establishment of this intestinal injury model was successful. A study by Li et al. demonstrated that *L. rhamnosus* GR-1 can induce PINK1/Parkin to mediate mitochondrial autoregulation, clear damaged mitochondria and decrease ROS production and NLRP3 inflammasome activation, decrease *E. coli*-induced apoptosis, and can decrease the levels of *IL-1β*, *IL-8*, *TNF-α* and other cytokines (Li et al., 2021). Fukuyama et al. found that *Lactobacillus acidophilus* CRL2074 and *L. rhamnosus* CRL2084 had a better regulatory effect, decreasing the relative expression of *IL-1β*, *IL-8*, and *CXCL3* (Fukuyama et al., 2020). In this experiment, LGG addition significantly decreased the relative expression of *IL-8* in the duodenum and *IL-1β* in the jejunum. The results of this trial are similar to the above results, indicating that LGG has the potential to enhance intestinal immunity.

### 4.6 The effect of LGG on the expression of liver function-related genes in LPS-stressed broiler chickens

The normal maintenance of the body’s immune function is conducive to improving the intestinal barrier function and the body’s resistance to diseases and infections. Based on previous research findings, LGG has a critical protective effect on the intestinal barrier and intestinal immune function, and then its effect on the liver was studied. The liver is the body’s most important metabolic and innate immune organ. Its abundant natural killer cells and macrophages are the main line of defense for natural immunity in the liver (Zhi et al., 2020). Most inflammatory responses are related to immune mechanisms, and macrophages are the key factor in initiating inflammation (Cho et al., 2021). Heat shock proteins are a type of highly conserved stress proteins. Hsp70 is the most highly conserved protein known so far. It has a protective effect on cells under stress conditions (such as ultraviolet radiation, bacterial infection, or inflammation), such as improving cell Injury tolerance and stress capacity (Jeyachandran et al., 2023). Lyu et al. found that LPS stimulation down-regulates Hsp70 and up-regulates the expression of proinflammatory mediators TNF-α and IL-6 induced by it, thereby inhibiting the activation of NF-κB in macrophages (Lyu et al., 2020). In this experiment, LPS caused oxidative stress and liver damage and significantly decreased the relative expression of *Hsp70* in the liver, indicating that LPS caused stress injury to broilers. Matrix metalloproteinase 9 (MMP9) is an important pathogenic factor in inflammatory diseases. High concentrations of MMP9 can lead to increased intestinal tight junction permeability *in vitro* or *in vivo* (Al-Sadi et al., 2019). Experiments have shown that the expression of *MMP9* in the damaged liver increases significantly when acute liver failure is induced by LPS, etc.; in addition, MMP9 also plays a certain role in acute hepatitis (Yan et al., 2008; Wielockx et al., 2001). In this experiment, LPS significantly increased the relative expression of *MMP9* in the liver. Escamilla et al. (2012) used LGG to reduce the infiltration, invasion, and metastasis capabilities of tumor cells by reducing the expression of *MMP9*, inhibiting the degradation of IκB, and increasing the expression levels of *IL-8* and *ZO-1* (Escamilla et al., 2012). Consistent with the above results, in this experiment, LGG addition significantly decreased the relative expression of *MMP9* in the liver; under LPS stimulation, LGG addition significantly decreased the relative expression of *MMP9* in the liver, indicating that LGG may have a liver protective effect.

## 5 Conclusion

This study found that LGG addition to the diet can improve the intestinal barrier and immune function under LPS challenge, and also has a slightly protective effect on the liver.

## Data Availability

The datasets presented in this study can be found in online repositories. The names of the repository/repositories and accession number(s) can be found in the article/supplementary material.
